# Role of miR-211 in Neuronal Differentiation and Viability: Implications to Pathogenesis of Alzheimer’s Disease

**DOI:** 10.3389/fnagi.2016.00166

**Published:** 2016-07-08

**Authors:** Chunying Fan, Qi Wu, Xiaoyang Ye, Hongxue Luo, Dongdong Yan, Yi Xiong, Haili Zhu, Yarui Diao, Wei Zhang, Jun Wan

**Affiliations:** ^1^Shenzhen Key Laboratory for Neuronal Structural Biology, Biomedical Research Institute, Shenzhen-Peking University–The Hong Kong University of Science and Technology Medical CenterShenzhen, China; ^2^Ludwig Institute for Cancer Research, La JollaCA, USA; ^3^Division of Life Science, The Hong Kong University of Science and TechnologyHong Kong, China

**Keywords:** microRNA-211-5p, NUAK1, neurite branching, neuronal viability, amyloid β (Aβ), Alzheimer’s disease (AD)

## Abstract

Alzheimer’s disease (AD) is an age-related irreversible neurodegenerative disorder characterized by extracellular β Amyloid(Aβ) deposition, intracellular neurofibrillary tangles and neuronal loss. The dysfunction of neurogenesis and increased degeneration of neurons contribute to the pathogenesis of AD. We now report that miR-211-5p, a small non-coding RNA, can impair neurite differentiation by directly targeting NUAK1, decrease neuronal viability and accelerate the progression of Aβ-induced pathologies. In this study, we observed that during embryonic development, the expression levels of miR-211-5p were down-regulated in the normal cerebral cortexes of mice. However, in APPswe/PS1ΔE9 double transgenic adult mice, it was up-regulated from 9 months of age compared to that of the age-matched wild type mice. Studies in primary cortical neuron cultures demonstrated that miR-211-5p can inhibit neurite growth and branching via NUAK1 repression and decrease mature neuron viability. The impairments were more obvious under the action of Aβ. Our data showed that miR-211-5p could inhibit cortical neuron differentiation and survival, which may contribute to the synaptic failure, neuronal loss and cognitive dysfunction in AD.

## Introduction

Neurogenesis, or the ability of the brain to generate new neurons, was thought originally to occur only during brain development in the fetus and young child (Nixon et al., 2010). Recently, the presence of postnatal neurogenesis events referred to as adult neurogenesis has been demonstrated. Few but critical differences have been reported between embryonic and adult forms of neurogenesis (Temple, 2001). In mammalian brains, adult neurogenesis is limited to specific brain regions, such as the hippocampal dentate gyrus and the subventricular zone/olfactory bulb system (Winner and Winkler, 2015). The rate of adult neurogenesis declines with age (Altman and Das, 1965; Altman and Das, 1966; He and Crews, 2007; Manganas et al., 2007). An important aspect regarding adult neurogenesis is its modulation resulting from a variety of genetic, epigenetic, and transcriptional factors as well as environmental factors, age, and acute and chronic diseases (Ma et al., 2010; Mu et al., 2010; Winner and Winkler, 2015). Impaired adult neurogenesis has been shown in numerous animal models of neurodegenerative diseases (Winner et al., 2011). Alterations in adult neurogenesis appear to be a common hallmark of different neurodegenerative diseases, indicating that in addition to the loss of mature neurons, the endogenous ability of the adult brain to renew neurons and the putative functions of these new neurons are also damaged or even lost (Winner and Winkler, 2015).

Alzheimer’s disease is the most common human neurodegenerative disease, with more than 30 million sufferers worldwide and is characterized by gradual memory loss, cognitive impairments, and deterioration of language skills (Zhou et al., 2011). The neurite atrophy and synaptic loss induced by β Amyloid (Aβ) are considered to be the major causes of gradual cognitive deterioration in AD (Masliah et al., 2001; Selkoe, 2002). Recently, more studies showed that impairment of adult neurogenesis occurred in AD brains much earlier than neuronal loss. Understanding the mechanisms of changes in neurogenesis due to AD might give some clues for AD diagnosis and treatment.

NUAK1 is a member of the novel (Nua) kinases family with the alternative names ARK5 or OMPHK1 (Bekri et al., 2014). A number of previous studies indicated that NUAK1 was involved in regulation of proliferation, promotion of cell survival, invasion, and metastasis, induction of senescence and loss of cellular adhesion (Suzuki et al., 2003; Hou et al., 2011; Lu et al., 2013; Sun et al., 2013; Bell et al., 2014; Shi et al., 2014). It has been reported that NUAK1 regulates both axon growth and branching involved in neurogenesis (Courchet et al., 2013), but little is known about its role in AD pathologies.

MiRNAs are small non-coding RNAs which mainly bind to the 3′ untranslated region (3′-UTR) of target mRNAs and induce mRNA degradation or the inhibition of translation (Lagos-Quintana et al., 2001). An individual miRNA can down-regulate hundreds of mRNA targets by interacting with partially complementary sequences within their 3′UTRs. Our previous study found that miR-211-5p was up-regulated in the cortexes of APPswe/PS1ΔE9 double transgenic mice, which are commonly used as an AD mouse model, suggesting the pivotal role of miR-211-5p in AD pathology (Luo et al., 2014). NUAK1 has been demonstrated to be one of the targets of miR-211-5p with three miR-211-5p binding sites in its 3′UTR (Bell et al., 2014). Studies have shown that miR-211-5p regulates cell proliferation, differentiation, survival, invasion, and metastasis (Asuthkar et al., 2012; Cai et al., 2012; Bell et al., 2014; Xia et al., 2015). However, there are no relative reports about the role of miR-211 in neuronal differentiation and neurodegenerative diseases at present.

In this study, we found that miR-211-5p regulated neurite outgrowth by targeting NUAK1. This pathway also plays an important role in aggravating Aβ-triggered neuronal cell impairment.

## Materials and Methods

### Animals

All procedures involving animal experiments were in accordance with animal use protocols approved by the Committee for the Ethics of Animal Experiments, Shenzhen-Peking University-The Hong Kong University of Science and Technology Medical Center (SPHMC; protocol number 2011-004). Double-transgenic mice (APPswe/PSΔE9) (held by The Jackson Laboratory, strain name: 117 “B6.Cg-Tg (APPswe, PSEN1ΔE9)85Dbo/Mmjax)” (Borchelt et al., 1996) and their corresponding WT controls (C57BL/6J mice) were purchased from the Model Animal Research Center of Nanjing University. ICR mice were purchased from Guangdong Medical Lab Animal Center. All the mice were raised in a temperature-controlled room with free access to food and water, and 12 h light–dark cycle. APPswe/PSΔE9 mice and their non-transgenic littermates, as well as ICR mice at different age stages were used in this study. The mice were anesthetized with pentobarbital (50 mg/kg). Once removed and dissected, the cortexes of the brains were frozen in TRIzol Reagent (Invitrogen) for RNA extraction or in liquid nitrogen for protein extraction.

### DNA Constructs and miRs

Mouse NUAK1 cDNA was amplified by PCR from image clone 30355405 (accession number BC082328; Open Biosystems) followed by insertion into PT-GFP vector (a modified vector from pEGFP-C1) by XhoI and EcoRI enzyme sites (Courchet et al., 2013). The mimic and inhibitor of miR-211-5p and controls were purchased from Ribo Bio Company.

### Cell Culture and Transfection

Neuro2A cells, a mouse neuroblastoma cell line, were cultured in DMEM (GIBCO) containing 10% fetal bovine serum (Hyclone), 50 U/ml penicillin and 50μg/ml streptomycin (GIBCO). Mouse primary cortical neurons from E18.5 mouse embryos were cultured on Poly-L-Lysine (PLL)-coated culture plates in Neurobasal medium containing B27 supplement, 0.05 mM glucose, and 0.5 mM L-glutamine. Cells were grown in a humidified incubator at 37°C with 5% CO_2_. For immunofluorescence, neurons were maintained on coverslips. Transfections were performed using Lipofectamine 2000 Reagent (Invitrogen) according to the manufacturer’s instructions in Neuro2A cells 12 h after plating. Primary cultured cortical neurons were transfected immediately after culturing or at DIV7.

### Preparation and Treatment of Aggregated Aβ1-42

Aβ1-42 (Sigma) was initially dissolved in Hexafluoroisopropanol (HFIP) to a final concentration of 1 mM. Next, HFIP was allowed to evaporate in the fume hood. HFIP-treated samples were completely re-suspended to 5 mM in dimethyl sulfoxide (DMSO) (Sigma) by pipette mixing followed by diluting to 0.1 mM in phosphate buffered saline (PBS). Next, the diluted Aβ1-42 was incubated at 37°C for overnight to induce aggregation.

### RNA Extraction and qPCR

The RNA for qRT-PCR was extracted from mouse cortexes through homogenate or cells using TRIzol reagent and then was reverse transcripted as described in the previous study (Xia et al., 2015). RNA expression levels were quantified either using TaqMan probe (RiboBio) qPCR analysis or iQTM SYBR^®^ Green Supermix (Bio-Rad)-based qPCR assays. Standard curves and normalization factors were used to calculate and normalize the absolute copies of miR-211-5p as previously described (Wang Y. et al., 2015). All qPCR reactions were carried out on a CFX96^TM^ Real-Time PCR detection system (Bio-Rad). Glyceraldehyde-3-phosphate dehydrogenase (GAPDH) was used as the endogenous control of mRNA while U6 snRNA was used as the housekeeping gene of miRNA. Relative expression was calculated by using the delta–deltaCt method. All the primer sequences are listed in **Table 1**.

**Table 1:** Sequence of primers and probes.

Primer/probe name	Sequence
U6-F	5′-GCTTCGGCAGCACATATACTAAAAT-3′
U6-R	5′-CGCTTCACGAATTTGCGTGTCAT-3′
MiR-211-5p-F	5′-GATCTTCCCTTTGTCATCC-3′
Has miR-R	5′-GTGTCGTGGAGTCGGCAA-3′
MiR-211-5p-RT	5′-GTCGTATCCAGTGCGTGTCGTGGAGTCGGCAATTGCACTGGATACGACAGGCAA-3′
MsGAPDH-F	5′-AACTTTGGCATTGTGGAAGG-3′
MsGAPDH-R	5′-GGATGCAGGGATGATGTTCT-3′
MsNUAK1-F	5′-GAGCCCACTCTATGCGTC-3′
MsNUAK1-R	5′-ATGTCCTCGATGGTGGCT-3′
MiR-211-5p probe	5′-FAM-CACTGGATACGACAGGCAAAGGAT-TAMRA-3′
U6 probe	5′-FAM-CCTTGCGCAGGGGCCATGCTAATC-TAMRA-3′

### Western Blot Analysis

Mouse cortical tissues or cultured cells were lysed in RIPA buffer [150 mM NaCl, 1% (v/v) Nonidet P-40, 0.5% deoxycholic acid, 0.1% (w/v) sodium dodecyl sulfate (SDS)] containing protease inhibitors (cocktail, Sangon) and 1 mM phenylmethanesulfonyl fluoride (Sigma) for 15 min. The lysates were centrifuged at 14000 × g for 10 min at 4°C. Protein samples (supernatants) were immediately transferred into new tubes, and the concentration was measured using Bradford Reagent (Sigma). After boiling for 10 min at 98°C, the samples were separated on SDS–polyacrylamide gel electrophoresis (PAGE) gels followed by transferring onto nitrocellulose membranes (PALL). The membranes were treated with the primary antibodies against NUAK1 (Cell Signaling Technology, rabbit polyclonal antibody) and GAPDH (Sangon, rabbit polyclonal antibody) overnight at 4°C following blocking with 5% (w/v) skim milk in TBS-T (Tris-buffered saline containing 0.1% Tween-20) for 1 h. Next, the membrane was washed with TBS-T buffer three times and incubated with infrared-labeled secondary antibodies (Odyssey, Goat anti-rabbit IR Dye @ 680 CW) at room temperature for 2 h. After washing, the membranes were finally scanned with Odyssey v3.0. The intensity of each band was quantified using Image J analysis.

### Cell Viability Assays

Cell viability was assessed by measuring formazan produced by 3-(4,5-dimethyl-2-thiazolyl)-2,5-diphenyl-2H-tetrazolium bromide (MTT). The amount of formazan is proportional to the number of viable cells. MTT reagent was added to primary cortical neurons with different treatments as indicated at 1/10 volume of the medium. Cells were incubated for 4 h at 37°C. The medium with MTT was removed and formazan crystals were dissolved in DMSO. The optical density was determined at 490 nm using a microplate reader (Bio-Rad). Cell viability was presented as percentage of the non-treated control.

### Immunofluorescence Imaging

Neurons were fixed for 30 min at room temperature in 4% (w/v) paraformaldehyde. PBS containing 0.4% Triton X-100, 1% bovine serum albumin (BSA) (Sigma), 4% Goat Serum was used for 30 min to permeabilize and block non-specific staining. Neurons were incubated overnight at 4°C with primary antibody (anti-β-tubulin III, 1:800, Sigma, mouse monoclonal antibody). After the incubation with the secondary antibody (anti-mouse, CY3, 1:500, Jackson Immuno Research) for 2 h at room temperature, the coverslips were mounted on slides with anti-fade-mounting medium (Beyotime). The fluorescent signals were observed using a fluorescence microscope. Length and branch numbers of the longest neurites were quantified with Image J analysis.

### Statistical Analysis

Data are expressed as the mean ± SEM for tissues or mean ± SD for cells. A Student’s *t*-test was utilized to perform the statistical analysis using SPSS 19.0 software. A difference was considered statistically significant if the *p* value was <0.05.

## Results

### MiR-211-5p Modulates NUAK1 Levels in Neuro2A Cells and Mouse Primary Cultured Cortical Neurons

**FIGURE 1:**
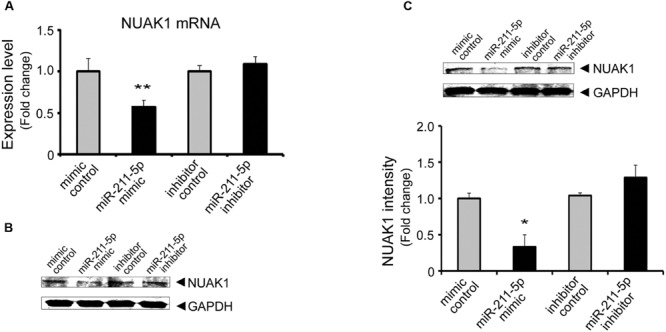
**MiR-211-5p regulates NUAK1 expression in Neuro-2a cells and primary mouse cortical neurons. (A)** and **(B)** miR-211-5p mimic (100 nM) or inhibitor (100 nM) was transfected into Neuro2A cells. After 24 h (RNA) to 48 h (protein), NUAK1 mRNA **(A)** or protein **(B)** levels were assessed by qRT-PCR or western blotting, respectively. The results were shown as the mean ± SD (^∗∗^*p* < 0.01). Three independent experiments were performed. **(C)** Western blot analysis of NUAK1 protein level in primary cortical neurons transfected with miR-211-5p mimic (1 μM) or inhibitor (1 μM) for 48 h. Image J software was used to quantify the gray degree values. The results were shown as the mean ± SD (^∗^*p* < 0.05). Three independent experiments were performed.

To determine whether NUAK1 is regulated by miR-211-5p in Neuro2A cells and primary cultured cortical neurons, we transfected cells with miR-211-5p mimic and inhibitor and then examined the NUAK1 mRNA and protein levels. Over-expression of miR-211-5p mimic resulted in a significant decrease of NUAK1 mRNA and protein levels. However, miR-211-5P inhibitor had no effect (**Figure 1**), indicating that additional unknown mechanisms may also be involved in NUAK1 regulation.

### MiR-211-5p Inhibits Neurite Growth and Branching via Targeting NUAK1

**FIGURE 2:**
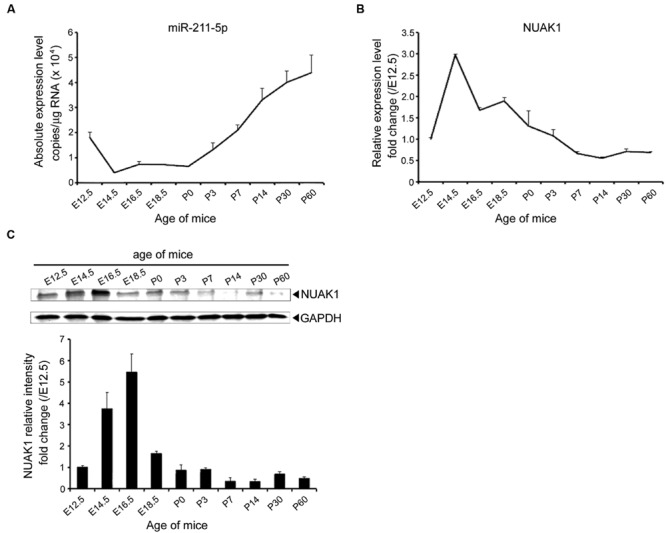
**Expression profile of miR-211-5p and NUAK1 in mice brains during development. (A)** The absolute copies of miR-211-5p in the cortexes of ICR mice during embryonic and postnatal development examined by TaqMan qRT-PCR were calculated and normalized using standard curves and normalization factors. The result was shown as the mean ± SEM (*n* = 5 for E12.5 to P7; *n* = 3 for P14 to P60). **(B)** Relative expression of NUAK1 mRNA examined by qRT-PCR in the cortexes during embryonic and postnatal development. The result was shown as the mean ± SEM (*n* = 2–5). **(C)** NUAK1 protein expression in the cortexes was examined by Western blotting during embryonic and postnatal development. Gray degree values are quantified by Image J software. The result was shown as the mean ± SEM.

To gain insight into the role of miR-211-5p in neurogenesis, qPCR, and Western blotting were performed to assess the expression levels of miR-211-5p and NUAK1 during mouse embryonic and postnatal cortex development. MiR-211-5p expression is down-regulated during the embryonic development after E12.5 followed by an increase after birth (**Figure 2A**). NUAK1 mRNA and protein are highly expressed from E12.5 to P0, which is in contrast to that of the miR-211-5p (**Figures 2B,C**).

**FIGURE 3:**
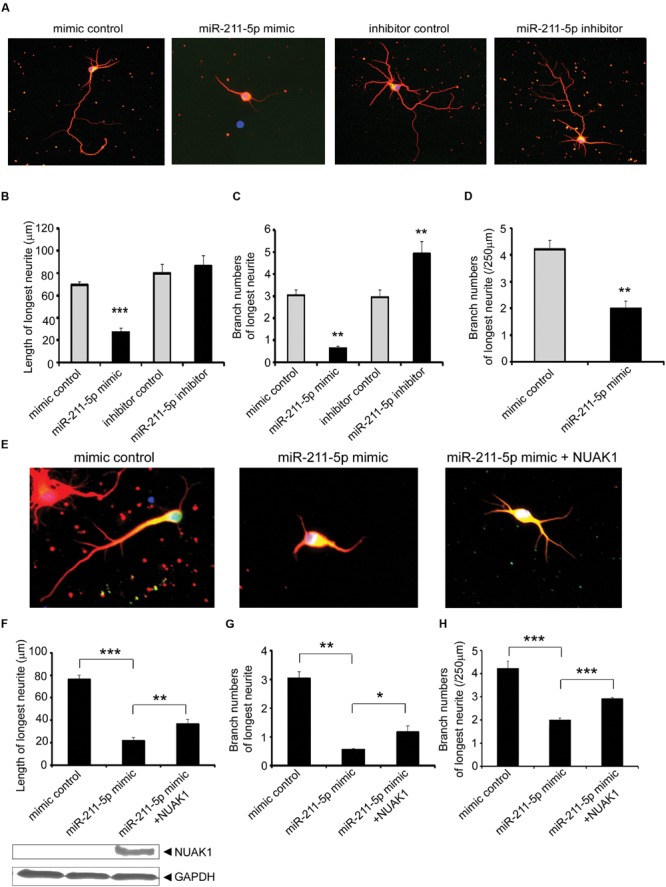
**MiR-211-5p inhibits neurite growth and branching via its target NUAK1. (A)** Mouse E18.5 primary cortical neurons were co-transfected with PT-GFP and miR-211-5p mimic or inhibitor (concentration ratio 1:3) before culturing. After 5 days of culture, the cells were fixed and immunostained with anti-tubulin III antibody. The longest neurite was observed and considered as the axon. **(B–D)** Quantitation of the longest neurite length or branches after overexpression or inhibition of miR-211-5p by Image J software. The results were shown as the mean ± SD (^∗^*p* < 0.05, ^∗∗^*p* < 0.01, and ^∗∗∗^*p* < 0.001). **(E)** Cortical neurons were transfected with miR-211-5p with or without NUAK1 plasmid and similar experiments were performed as described in **(A)**. **(F–H)** Quantitation of the longest neurite length or branches by Image J software. The results were shown as the mean ± SD (^∗^*p* < 0.05, ^∗∗^*p* < 0.01, and ^∗∗∗^*p* < 0.001).

During early neuronal differentiation cultured *in vitro*, axons grow faster than dendrites and we focused our study on the longest neurite and considered it as an axon. After the transfection of miR-211-5p mimic or inhibitor, we found that neurons with the overexpression of miR-211-5p displayed significantly decreased growth and branching of longest neurite, whereas miR-211-5p inhibitor induced markedly increased branching but without the length change (**Figures 3A–C**). In order to examine whether the less-branched neurites could be a secondary result of shorter neurites, the neurite number was normalized for by neurite length. The result showed that the longest neurite branching is still significantly reduced when miR-211-5p was overexpressed (**Figure 3D**). As NUAK1 is one of the targets of miR-211-5p, we further determined whether NUAK1 could alleviate the insult of miR-211-5p on neurite length and branching in cortical neurons. It was confirmed that overexpression of NUAK1 could rescue miR-211-5p mimic-induced neurite impairment (**Figures 3E–H**), indicating that miR-211-5p inhibits both neurite growth and branching by regulating NUAK1.

### MiR-211-5p-NUAK1 Pathway Is Involved in Alzheimer’s Disease Pathologies

**FIGURE 4:**
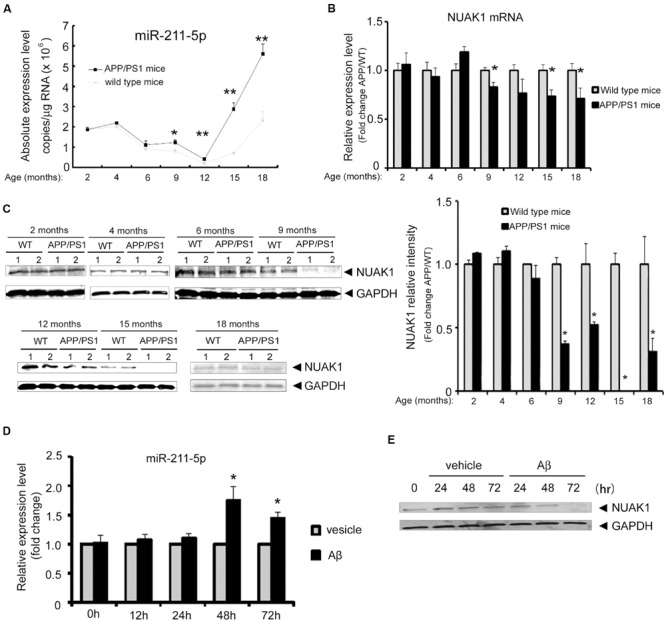
**Expression profile of miR-211-5p and NUAK1 in APP/PS1 mice cortexes and Aβ-treated primary cortical neurons. (A)** The absolute copies of miR-211-5p in WT and APP/PS1 mice cortexes were examined by TaqMan qRT-PCR (five pairs of mice at each stage). The data were calculated and normalized using standard curves and normalization factors. The result was shown as the mean ± SEM (^∗^*p* < 0.05, ^∗∗^*p* < 0.01, and *n* = 6). **(B)** Relative expression levels of NUAK1 in the same mice cortexes as described in **(A)** by qRT-PCR analysis. The result was shown as the mean ± SEM (^∗^*p* < 0.05, ^∗∗^*p* < 0.01, and *n* = 5). **(C)** NUAK1 protein levels in WT and APP/PS1 mice cortexes were examined by western blotting. Glyceraldehyde-3-phosphate dehydrogenase (GAPDH) is used as a reference gene. Gray degree values are quantified by Image J software. The result was shown as the mean ± SEM. **(D,E)** Mouse primary cortical neurons at DIV7 were treated with Aβ1-42 (1 μM) for different time intervals as indicated. MiR-211-5p expression was quantified by semi-quantitative RT-PCR with SYBR Green **(D)**. The result was shown as the mean ± SD (^∗^*p* < 0.05, ^∗∗^*p* < 0.01). NUAK1 protein expression was examined by Western blotting **(E)**.

To explore the dynamic changes of miR-211-5p and NUAK1 expression during the development of AD pathology, we examined their expression levels in the cortexes of APP/PS1 and WT mice with ages spanning from 2 to 18 months by real-time quantitative PCR using TaqMan probe. MiR-211-5p expression was significant higher in APP/PS1 mice than that of WT mice beginning at 9 months of age (**Figure 4A**). However, NUAK1 mRNA and protein levels have the opposite expression patterns (**Figures 4B,C**). To further confirm their results in a cell model, mouse cortical neurons were treated with Aβ1-42 at DIV7 for different intervals. MiR-211-5p and NUAK1 RNA levels were detected by semi-quantitative RT-PCR using SYBR Green and the protein levels were examined by Western blotting. The dynamic changes of miR-211-5p and NUAK1 were consistent with that from mice tissues (**Figures 4D,E**), indicating that miR211-5p-regulated NUAK1 pathway may have a relationship with the pathological process of AD.

### MiR-211-5p Aggravates the Insult of Aβ on Neurite Growth and Branching

**FIGURE 5:**
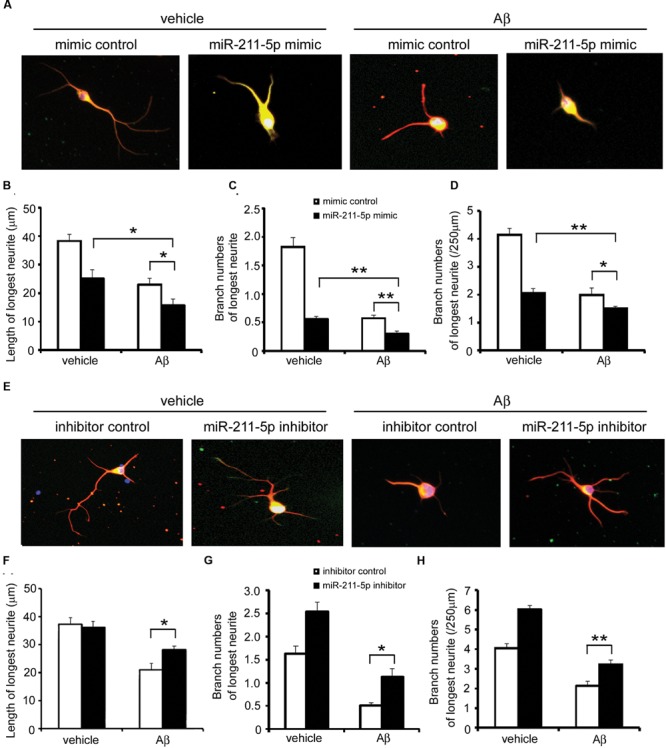
**miR-211-5p aggravates the insult of Aβ on neurite growth and branching. (A)** Mouse primary cortical neurons were transfected with PT-GFP and miR-211-5p mimic (500 nM) before culturing. Next, the cells were cultured for 3 days followed by Aβ1-42 treatment (7 μM) for 48 h. Neurites were immunostained with anti-β-tubulin III antibody. **(B–D)** The longest neurite length and branch numbers were quantified of by Image J software. The results were shown as the mean ± SD (^∗^*p* < 0.05, ^∗∗^*p* < 0.01). **(E)** Mouse primary cortical neurons were transfected with PT-GFP and miR-211-5p inhibitor (1 μM) and the same experiment was performed as described in **(A)**. **(F–H)** The longest neurite length and branch numbers were quantified by Image J software. The results were shown as the mean ± SD (^∗^*p* < 0.05, ^∗∗^*p* < 0.01).

To determine whether the effect of miR-211-5p is involved in Aβ1-42 impairment, we overexpressed miR-211-5p mimic or inhibitor in cultured cortical neurons followed by Aβ treatment. Overexpression of miR-211-5p mimic dramatically aggravated the longest neurite length and branching damage induced by Aβ (**Figures 5A–D**). However, miR-211-5p inhibitor protected neurons from Aβ damage (**Figures 5E–H**).

### MiR-211-5p Increases Aβ Cytotoxicity in Neurons

**FIGURE 6:**
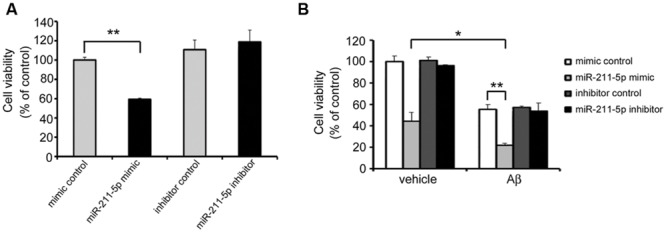
**miR-211-5p increases Aβ cytotoxicity on neuron viability. (A)** Mouse primary cultured cortical neurons were transfected with miR-211-5p mimic or inhibitor at DIV7. Twenty-four hours later, the 3-(4,5-dimethyl-2-thiazolyl)-2,5-diphenyl-2H-tetrazolium bromide (MTT) assay was performed to evaluate the cell viability. **(B)** Mouse primary cultured cortical neurons were transfected with miR-211-5p mimic or inhibitor at DIV7 for 24 h followed by Aβ1-42 treatment (10 μM) for another 24 h. Next, the MTT assay was performed to evaluate the cell viability. The results were shown as the mean ± SD (^∗^*p* < 0.05, ^∗∗^*p* < 0.01).

In order to examine the role of miR-211-5p on neuron viability, an MTT assay was performed after miR-211-5p mimic or inhibitor transfection. The viability of primary cultured cortical neurons was dramatically decreased with the overexpression of miR-211-5p, whereas miR-211-5p inhibitor had no effect (**Figure 6A**). When Aβ was treated together with miR-211-5p overexpression, the cell death was further induced, but miR-211-5p inhibitor showed no effect (**Figure 6B**).

## Discussion

Adult neurogenesis involves several crucial steps of neural development and requires several processes to produce novel neurons: neural stem cell proliferation, cell differentiation, cell migration, and cell survival and/or integration, and each of these processes is regulated independently (Nixon et al., 2010). NUAK1 is highly expressed in the cortex during embryonic development and promotes axon extension and branching, affecting neuronal differentiation (Courchet et al., 2013), which might be a potential therapeutic approach for the repair of ischemic stroke (Wang Z. et al., 2015). However, the role of NUAK1 mediated neuronal differentiation in AD pathologies is unknown. Previous studies have indicated that synaptic loss and neuronal death caused by abnormal deposition of Aβ are the major cause of cognitive deterioration in AD (Hardy and Selkoe, 2002; Kayed et al., 2003). Neurogenesis plays a key role in the rescue of impaired neurons and improvement of cognitive decline in AD mice (Bruel-Jungerman et al., 2007; Deng et al., 2010). Our results showed that miR-211-5p was up-regulated and NUAK1 was down-regulated in both AD cell and mouse models. Although our data showed NUAK1 protein levels go down at 15 months of age and come back at 18 months old, we do not know the significance of this and need more examination to avoid individual difference. MiR-211-5p/NUAK1 was one of the pathways induced by Aβ to damage neurogenesis, which was consistent with the previous studies regarding impaired adult neurogenesis in AD transgenic models (Winner et al., 2011; Winner and Winkler, 2015).

MiRNAs modulate cellular regulatory processes via their ability to alter gene expression. Recent accumulating evidence has implicated the dysregulation of miRNAs expression in AD (Luo et al., 2014; Lei et al., 2015; Santa-Maria et al., 2015; Ye et al., 2015; Zhu et al., 2015). Some studies have identified miRNAs that are involved in AD by regulating neurogenesis. The amyloid precursor protein intracellular domain (AICD) processed from APP binds to regulatory regions of specific miRNAs in the human genome and suppresses neuronal differentiation through transcriptional regulation of miRNAs, and thus results in decreased neurogenesis (Shu et al., 2015). MiR-206 regulates brain-derived neurotrophic factor which promotes neurogenesis in an AD model (Lee et al., 2012). These findings demonstrate the interesting link among miRNA, neurogenesis and AD. In this study, we first combined the role of miRNA in embryonic brain development and in AD pathologies. MiR-211-5p was not only involved in neuronal differentiation at embryonic stage, but also involved in Aβ-induced pathologies in an AD mouse model by targeting NUAK1. The mechanism of miR-211-5p increase induced by Aβ is still unknown, but our data shows that the destructive role of miR-211-5p on neurogenesis participates in the Aβ impairment of neurons. Although we found miR-211-5p inhibitor could not affect NUAK1 expression, miR-211-5p inhibitor alleviated the insult of Aβ on neurite growth and branching in our study. However, there is no effect of miR-211-5p inhibitor on neuron viability, indicating that there are more factors involved in this complicated process, such as bcl-2 (An et al., 2014).

Our work indicated that miR-211-5p may play a role in AD development by affecting neurogenesis and neuronal viability. As miRNAs play roles as a network during different processes, the importance of miR-211-5p in Aβ-related pathologies still needs detailed elucidation. We still need more animal experiments to confirm the role of miR-211-5p further in AD. For example, miR-211-5p knockout mice will be crossed with APP/PS1 mice to further confirm the role of miR-211-5p in Aβ-induced neurogenesis impairment. However, our study supports the translational potential of miR-211-5p in AD diagnosis and treatment in the future.

## Author Contributions

CF, QW, WZ, and JW conceived and designed the experiments. CF, QW, XY, HL, DY, YX, and HZ performed the experiments. CF, QW, YD, WZ, and JW analyzed the data. CF, WZ, and JW wrote the paper.

## Conflict of Interest Statement

The authors declare that the research was conducted in the absence of any commercial or financial relationships that could be construed as a potential conflict of interest.

## Abbreviations

AD: Alzheimer’s disease
Aβ: β amyloid peptide
miRNA: microRNA
APP: amyloid precursor protein
DIV: days *in vitro*
NPC: neural progenitor cells
PS1: presenilin 1
WT: wild type
